# Proteome Profiling in Murine Models of Multiple Sclerosis: Identification of Stage Specific Markers and Culprits for Tissue Damage

**DOI:** 10.1371/journal.pone.0007624

**Published:** 2009-10-28

**Authors:** Ralf A. Linker, Peter Brechlin, Sarah Jesse, Petra Steinacker, D. H. Lee, Abdul R. Asif, Olaf Jahn, Hayrettin Tumani, Ralf Gold, Markus Otto

**Affiliations:** 1 Department of Neurology, St. Josef-Hospital, Ruhr-University, Bochum, Germany; 2 Department of Neurodegeneration and Restorative Research, Center of Neurological Medicine, University of Goettingen, Goettingen, Germany; 3 DFG Research Center for Molecular Physiology of the Brain, Goettingen, Germany; 4 Department of Neurology, University of Ulm, Ulm, Germany; 5 Department of Clinical Chemistry, University of Goettingen, Goettingen, Germany; 6 Max-Planck-Institute for Experimental Medicine, Goettingen, Germany; Julius-Maximilians-Universität Würzburg, Germany

## Abstract

The identification of new biomarkers is of high interest for the prediction of the disease course and also for the identification of pathomechanisms in multiple sclerosis (MS). To specify markers of the chronic disease phase, we performed proteome profiling during the later phase of myelin oligodendrocyte glycoprotein induced experimental autoimmune encephalomyelitis (MOG-EAE, day 35 after immunization) as a model disease mimicking many aspects of secondary progressive MS. In comparison to healthy controls, high resolution 2 dimensional gel electrophoresis revealed a number of regulated proteins, among them glial fibrilary acidic protein (GFAP). Phase specific up-regulation of GFAP in chronic EAE was confirmed by western blotting and immunohistochemistry. Protein levels of GFAP were also increased in the cerebrospinal fluid of MS patients with specificity for the secondary progressive disease phase. In a next step, proteome profiling of an EAE model with enhanced degenerative mechanisms revealed regulation of alpha-internexin, syntaxin binding protein 1, annexin V and glutamate decarboxylase in the ciliary neurotrophic factor (CNTF) knockout mouse. The identification of these proteins implicate an increased apoptosis and enhanced axonal disintegration and correlate well the described pattern of tissue injury in CNTF −/− mice which involve oligodendrocyte (OL) apoptosis and axonal injury.

In summary, our findings underscore the value of proteome analyses as screening method for stage specific biomarkers and for the identification of new culprits for tissue damage in chronic autoimmune demyelination.

## Introduction

The recent years faced substantial advance in early and at the same time accurate diagnosis of multiple sclerosis (MS, [1], [2]). Yet, the need for reliable markers of disease prognosis is still unmet. While magnetic resonance imaging (MRI) has been proven useful in the early disease phases, the present routine MRI protocols are only of limited value for the prediction of long term prognosis of MS [3]. Thus, stage specific prognostic biomarkers are of high interest for accurate patient counseling. Still, evidence on the reliability of some present-day biomarkers is conflicting (for review see [4], [5]). While first data pointed at the value of serum anti-myelin antibodies as predictor of clinically definite MS, this could not be reproduced in follow-up studies [6], [7]. Some studies identified the intrathecal synthesis of oligoclonal IgM as predictor of an aggressive course of MS [8], [9], while it does not predict an early diagnosis [10]. Screening for polymorphisms associated with disease progression led to the identification of variations in the gene of the signaling molecule PD1 as genetic modifier [11] and a genome wide association studies identified the IL2RA and IL7RA as risk factors for MS [12]. For further systematic identification of candidate genes, expression profiling in MS may prove useful [13]–[15]. In the recent years, a vast number of gene array studies in MS were carried out and mainly allowed insights in therapeutic mechanisms or were employed to determine the response to immunomodulatory treatment (for example [16]). In contrast, only few studies addressed the systematic identification of regulated proteins so far [17]. In fact, protein profiling mainly focused on the screening for autoantibodies in autoimmune diseases [18].

Best candidates for prognosis relevant proteins may be those directly derived from the central nervous system (CNS) as target tissue of the immune reaction. Since the ready availability of these proteins in MS may prove difficult, it seems worthwhile to first investigate their regulation in experimental models. In particular, myelin oligodendrocyte glycoprotein induced experimental autoimmune encephalomyelitis (MOG-EAE) mimics many features of relapsing-progressive or secondary progressive MS [19], [20]. Indeed, gene expression profiling revealed differential gene regulation in the CNS as well as the microvascular compartment of EAE diseased mice [21], [22]. Recently, the differential expression of proteins in the inflamed spinal cord was also described by proteomic analysis using isobaric tags [23] and in correlation to immunohistochemistry [24]. While all these studies showed the technical feasibility of genomic or proteomic analyses in autoimmune demyelination, a candidate marker protein which could be directly transferred from the experimental screening to the clinical situation has not been described so far.

Here we employed proteome profiling of MOG-EAE and the ciliary neurotrophic factor knockout mouse (CNTF −/− mouse) as model with enhanced tissue injury including axonal degeneration and oligodendrocyte (OL) apoptosis to screen for disease regulated proteins which could serve as candidate biomarkers in MS patients.

## Materials and Methods

### Animals

C57BL/6 mice were purchased from Harlan (Borchen, Germany) and kept at the animal care facilities of the Institute for MS Research, University of Göttingen, Germany. CNTF −/− mice were backcrossed to the C57BL/6 background for more than 14 generations and bred at the in-house animal care facilities. These mice were characterized in detail in previous studies and display an increased OL apoptosis and enhanced axonal degeneration over the later phases of MOG-EAE [25], [26]. All animal experiments were performed in accordance with the Lower Saxony State regulations for animal welfare.

### Induction and clinical evaluation of active MOG-EAE

For active induction of EAE, mice received a s.c. injection at flanks and tail base of 200 µg MOG 35–55 peptide (Charité, Berlin, Germany) in PBS emulsified in an equal volume of CFA containing Mycobacterium tuberculosis H37RA (Difco, Detroit MI, USA) at a final concentration of 1 mg/ml. Two injections of pertussis toxin (List Biochemicals, Campbell, CA, USA, 400 ng per mouse i.p.) were given at the time of immunization and 48 hours later. Animals were weighed and scored for clinical signs of disease on a daily basis using a clinical score as described previously [25].

### Tissue processing

On day 35 or 37 after immunization (p.i.), mice were anesthetized with Ketanest (Inresa, Freiburg, Germany) and Rompun (Bayer, Leverkusen, Germany), transcardially perfused with saline and spinal cords were carefully removed. For histochemical analyses perfusion with saline was followed by a solution of 4% paraformaldehyde (PFA) and spinal cord tissue was postfixed in the same fixative for two hours. Subsequently, axial paraffin sections (3 µm) or, after resin embedding, semi-thin sections (0.5 µm) were obtained.

### Immunohistochemistry and histopathology

Histopathological evaluation included anti-GFAP immunohistochemistry (rat anti-GFAP, dilution 1∶1000, DAKO) and toluidine blue staining of semi-thin sections. For GFAP staining, the primary antibody was incubated overnight at 4°C. After washing, sections were incubated with the appropriate secondary antibody (Invitrogen Hamburg, Germany) at a dilution of 1∶100) and developed using diaminobenzidine (DAB) as chromogenic substrate. Specificity of staining was confirmed by omitting the primary antibody. Sections were mounted with Entellan (Sigma) and analyzed by light microscopy (Olympus, Hamburg, Germany).

### Two-dimensional difference gel electrophoresis (2D DIGE)

First-dimension isoelectric focusing was performed with immobilized pH 3 to 10 nonlinear gradient gels (24-cm Immobiline dry strips pH 3–10 NL; GE Healthcare). IPG strips were rehydrated for 24 h in 7 M urea−2 M thiourea−4% CHAPS−1% dithiothreitol (DTT)−0.4% IPG buffer pH 3–10 NL−0.002% bromophenol blue. Whole spinal cord samples were dissolved in 3 volumes of ice-cold acetone and precipitated overnight at −20°C. Proteins were pelleted, air dried for 1 h, and lysed in 7 M urea−2 M thiourea−4% CHAPS-30 mM Tris-HCl (pH 8.1) by rocking for 1 h at ambient temperature for subsequent labeling. Insoluble material was removed by centrifugation. The proteins were labeled as specified by the manufacturer with fluorescent dyes specifically developed for the two-dimensional 2D difference gel electrophoresis system (2D-DIGE) (CyDyes Cy2, Cy3, and Cy5 [GE Healthcare]). For the mixed internal standard, aliquots of each individual sample included in the experiment were pooled and labeled with Cy2 in the same dye-to-sample ratio. After 30 min incubation at 8°C in the dark, the labeling reaction was abrogated by adding 20 nmol lysine and incubating for further 10 min. The labeled samples were combined and diluted 1.33 fold by a stock solution containing 7 M urea, 2 M thiourea, 4% CHAPS, 4% IPG-buffer 4–7, 4% DTT w/v for subsequent IEF. Labeled samples were cup-loaded near the anodic end, and isoelectric focusing was carried out for a total of 56.000 Vh (1 h 150 V, ramp for 3 h to 300 V, ramp for 6 h to 1000 V, ramp for 3 h to 8000 V, hold at 8000 V for 4∶40 h). After focusing, the strips were equilibrated for two 25-min intervals in 6 M urea-125 mM Tris-HCl (pH 7.85)−3% SDS-20 (vol/vol) glycerol; 1% DTT was added for the first equilibrium step, and 4.2% iodoacetamide (IAA) was added for the second equilibrium step. Second dimension SDS-PAGE was performed with homogeneous 11% polyacrylamide gels (254 by 200 mm) by the method of Tastet et al. [27] with 150 g Tris/0.6 M HCl as gel buffer and taurine instead of glycine as buffering ion in the running buffer at 4 W/gel overnight at 25°C. The fluorescence signals of the three differently CyDye-labeled protein samples were imaged using a laser scanner recording band pass filtered emission wavelengths of 520 nm (Cy2); 580 nm (Cy3) and 670 nm (Cy5) respectively (Typhoon 9400 GE Healthcare). For comparison of wild type mice with or without EAE a set of 2 gels were run in a dye swap manner. For the comparison of CNTF knockout mice with wild-type controls, a set of 6 gels were run, with a total of 6 individual samples per group. The gels were analyzed with the different software modules of the DeCyder differential analysis software (GE Healthcare). Proteins were post-stained with colloidal Coomassie. Spots of interest were excised manually as described previously [28] and subjected to mass spectrometric protein identification

### Protein identification

Proteins were identified by two approaches as described recently [29], [30]. Briefly, an automated platform was used to digest the proteins in-gel with trypsin and to prepare the proteolytic peptides for MALDI-TOF-MS. For each sample, a peptide mass fingerprint (PMF) spectrum and fragment ion spectra of up to four selected precursor ions were acquired within the same automated analysis loop using an Ultraflex I mass spectrometer (Bruker Daltonics). Data base searches were performed with the Mascot Software 2.0 (Matrix Science) licensed in-house. Only proteins represented by at least one peptide sequence above significance threshold in combination with the presence of at least four peptide masses assigned in the PMF were considered as identified (for detailed reference see [31], [32]).

### Protein Sequence Analysis by LC-MS/MS

To confirm the data obtained from mass finger print analysis, some samples were subjected to peptide sequence analysis. The peptide sequencing analysis was performed as described elsewhere [29], [33]. Briefly, extracted peptides were dissolved in 0.1% formic acid (FA) and one microliter of sample was introduced using a CapLC autosampler (Waters, Manchester, UK) onto a µ-precolumn Cartridge (C18 pepMap, 300 µm×5 mm; 5 µm particle size, LC Packings Idstein, Germany) and further separated through a C18 pepMap100 nano Series (75 µm×15 cm; 3 µm particle size) analytical column (LC Packings Idstein, Germany). The mobile phase consisted of solution A (5% ACN in 0.1% FA) and solution B (95% ACN in 0.1% FA). The total sample running time was set to 60 min. Peptide sequencing was performed on a Q-TOF Ultima Global (Waters) mass spectrometer equipped with a nanoflow ESI Z-spray source in the positive ion mode. Multiple charged peptide parent ions were automatically marked and selected in the quadrupole and fragmented in the hexapole collision cell, and their fragment patterns were analyzed by time-of-flight. Data were acquired using MassLynx (v 4.0) software (Waters) on a Windows NT PC, and were further processed on a Protein-Lynx-Global-Server (PLGS) v. 2.1. (Waters, Manchester, UK). The raw data files were deconvoluted and deisotoped using the Max Ent lite algorithm (Waters). A PLGS module was used to generate Mascot-searchable *.pkl files. The *.pkl processed data were searched against a NCBI database via a Mascot search engine using a peptide mass and MS/MS tolerance of 0.5 Da. The search criteria were set with one missed cleavage by trypsin allowed and protein modifications set to methionine oxidation and carbamidomethylcysteine when appropriate.

### GFAP Western Blotting

Separation of proteins (homogenates 40 µg/lane), blotting, detection, and quantification of the signals were performed as described [34]. Blots were incubated with rabbit anti-GFAP antibody (DAKO, 1∶7500 in 3% MMP/TBST) followed by incubation with a biotylinated anti-rabbit antibody (Vector, 1∶10000). The signals were standardized relative to the amount of GFAP in wild-type spinal cord. A representative experiment of two independent analyses is shown (n* = *3 for each genotype).

### Patients

All analyses were approved by the local ethics committee of the University Ulm. Patients were admitted to the Department of Neurology, University Ulm in 2007. CSF-samples of 22 patients were collected within the scope of the routine work-up and were stored at −80°C until the carrying out of the laboratory experiments.

The MS patient group consists of 14 adult patients (age between 19 and 55) who were diagnosed with MS according to the McDonald criteria. Additionally, all patients were classified according to the EDSS score and underwent magnetic resonance imaging (MRI) of the neurocranium and spine. All investigated CSF-samples of MS patients showed oligoclonal bands. At the time of the lumbar puncture, the relapsing remitting MS patients did not receive any disease modifying treatment. In contrast, two patients suffering from secondary-chronic progressive MS received interferon-β therapy.

In the control group, eight adult patients (age range between 18 and 44 years) underwent lumbar puncture as a part of the neurologic differential diagnosis. None of these patients presented a chronic inflammatory neurologic disease. The CSF evaluations of these patients were assessed as normal findings. On the occasion, no oligoclonal bands were detected. Both, the cell count and the protein levels were in normal ranges.

### Measurement of the GFAP concentrations in CSF samples

Determination of GFAP concentration in the individual samples was performed according to the manufacturer protocol using an enzyme-linked immunoabsorbent assay (ELISA; BioVendor). The analytical limit of detection was 0.033 ng/ml. The sensitivity of the used assay takes the dilution of the samples into consideration and is calculated according to the formula: Assay sensitivity  =  Analytical limit of detection x sample dilution  = 0.033 ng/ml×3 = 0.1 ng/ml.

### Statistical Analysis

The comparison of the GFAP concentrations between the different subgroups was based on nonparametric rank tests (for two groups, Wilcoxon Mann-Whitney-U-test; for more than two groups, the Friedman 2-way ANOVA by ranks). Differences were considered significant at *p<0.05, and ** p<0.01.

## Results

### Proteome profiling in the chronic phase of murine MOG-EAE

**Figure 1:**
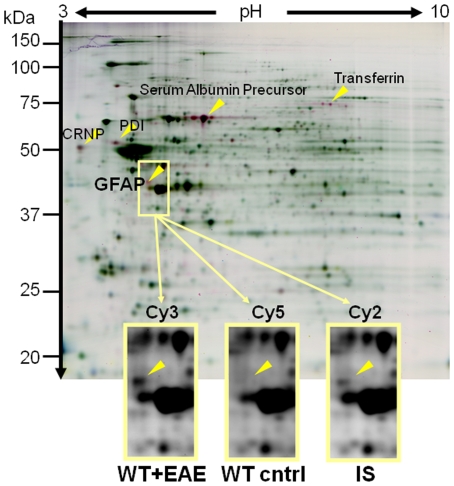
Proteome analysis of chronic MOG-EAE. 2D-DIGE gel of brain proteins from wild type mice with EAE, labeled with Cy3 (shown in red) versus healthy control (without EAE) labeled with Cy5 (shown in green). Images of gel region containing the glial fibrillary acidic protein (GFAP; identified by Mass Spectrometry) is shown in detail for the respective CyDye. IS  =  Internal Standard (Mixture of Samples). Proteins were separated on 24 cm IPG Strip pH 3–10 non-linear. CRNP =  Calreticulin Precursor; PDI = Protein Disulfid Isomerase Precursor.

To identify markers of disease progression in chronic progressive autoimmune demyelination, we investigated the regulation of proteins by proteome profiling of the early chronic phase (day 35 p.i.) of MOG-EAE in the C57BL/6 mouse as a model disease. 2DIGE of spinal cord tissue revealed a number of regulated proteins, among them the astrocyte marker glial fibrillary acidic protein (GFAP, see Fig. 1), but also transferrin precusor, calreticulin precursor and protein disulfide isomerase precursor (Table 1).

**Table 1:** 

Spot No.	Protein name	Swiss-Prot accession	MW [Da]	pI	coverage [%]	score	Peptides
1	Glial fibrillary acidic protein	Q80VX6	49870	5.27	45	731	22
2	Protein disulfide-isomerase precursor	P09103	57108	4.79	16	134	7
3	Protein disulfide-isomerase A3 precursor	P27773	56643	5.88	26	291	14
4	Serum albumin precursor	P07724	68648	5.75	45	760	21
5	Serotransferrin precursor	Q63915	76674	6.94	22	284	15

### Confirmation of GFAP up-regulation in the spinal cord of chronic MOG-EAE

**Figure 2:**
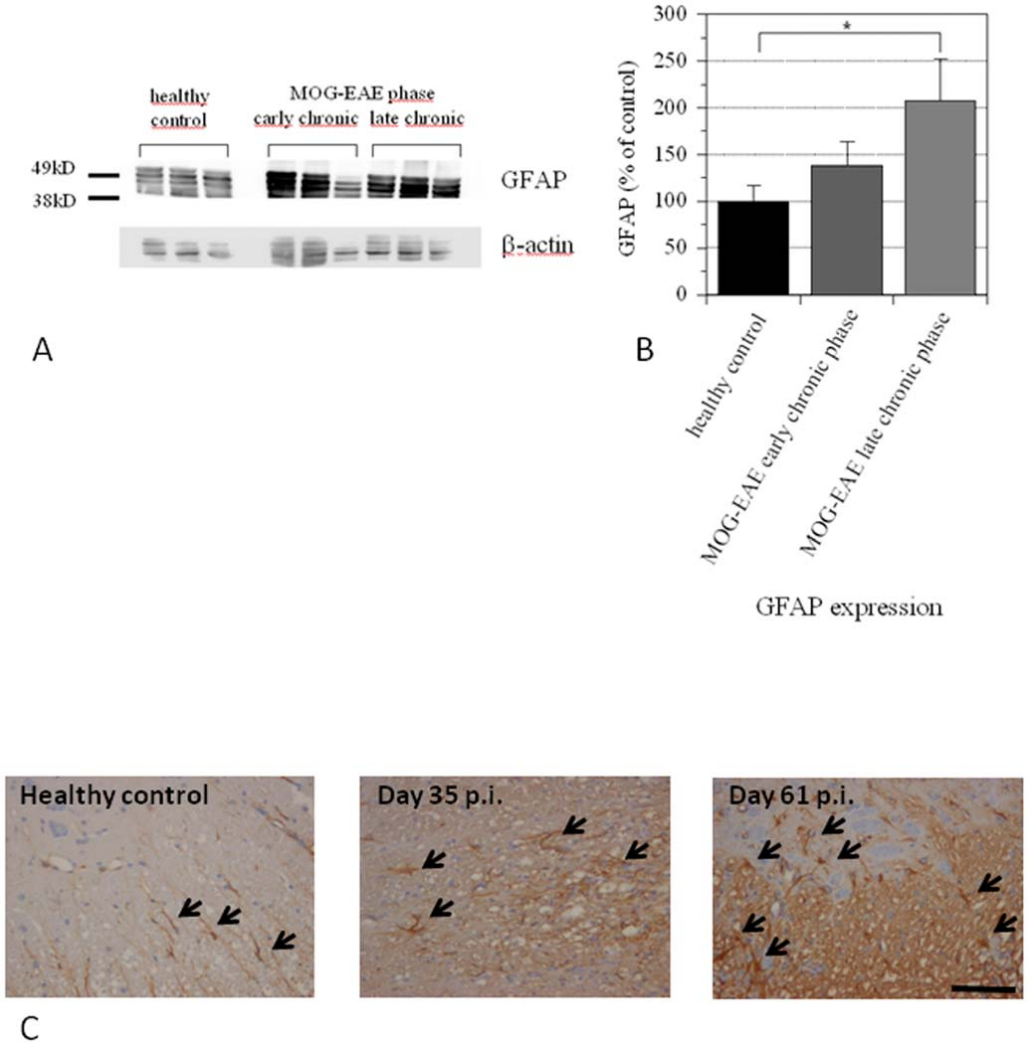
Up-regulation of GFAP over the course of MOG-EAE. (A) Western-Blot analysis of spinal cord protein preparation at the early chronic (day 35 p.i.) and late chronic phase of the disease (day 60 p.i.). Each lane represents the GFAP expression of one single mouse, β-actin serves as a loading control. (B) Quantification of optical densities of the GFAP labeling as shown in figure 2A. There was a clear increase in GFAP expression over the course of MOG-EAE (p<0.05 on day 60 p.i.). (C) Immunohistochemistry for GFAP in naïve C57BL/6 mice without EAE (left) and on day 35 p.i. (middle) and 60 p.i. (right). Representative images from spinal cord cross sections are shown, arrows indicate GFAP positive astrocytes which appear elongated in naïve mice and swollen in the chronic phases of MOG-EAE. Bar represents 20 µm.

We aimed to confirm GFAP as a regulated marker protein in murine MOG-EAE. To that end, protein levels of GFAP were investigated by Western Blot (Fig. 2) over the course of the disease at an early chronic (day 35 p.i.) and late chronic time point of MOG-EAE (day 60 p.i.). Quantification of protein levels revealed a significant, up to 2 fold increase of GFAP protein levels in the later disease phases (Fig. 2B).

These data were correlated with GFAP immunohistochemistry which revealed thin GFAP positive astrocytes without gliosis in naive control mice while over the course of MOG-EAE, astrocytes displayed strongly GFAP positive stellar shaped cell bodies accompanied by a diffuse GFAP staining indicating fibrillary gliosis (Fig. 2C).

### Regulation of GFAP in the CSF of MS patients

**Figure 3:**
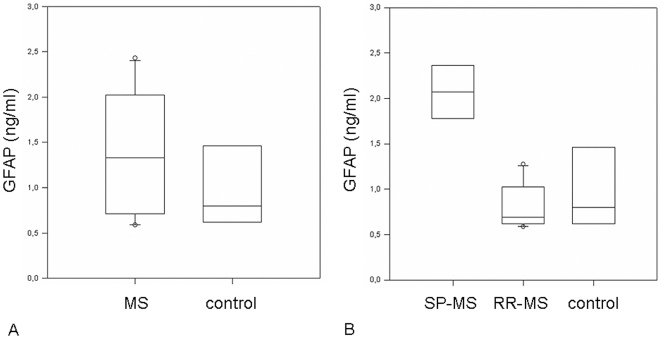
Detection of GFAP in CSF samples of secondary progressive MS patients. (A) Boxplot analysis of a group of unselected MS patients in comparison to control patients without neurological disease. GFAP levels in the CSF are increased in the multiple sclerosis (MS) group (p<0.05). (B) Boxplot analysis comparing secondary progressive MS (SP-MS) patients, relapsing remitting MS (RR-MS) patients and controls. GFAP levels in the CSF are exclusively increased in the secondary progressive MS group.

To prove the relevance of GFAP as a regulated protein in human autoimmune demyelination, we investigated this candidate marker in the CSF of MS patients. There was a significant increase of GFAP protein in patients suffering from secondary progressive MS, whereas patient with a relapsing remitting disease course displayed levels similar to healthy controls (see Table 2 for baseline characteristics of patients and Fig. 3A,B for results). The GFAP levels for secondary chronic progressive MS patients were between 1.7 ng/ml to 2.4 ng/ml (median 2.05 ng/ml) and in the relapsing remitting group between 0.587 ng/ml to 1.275 pg/ml (median 0.815 ng/ml). In the control group, patients displayed levels between 0.507 ng/ml to 1.595 ng/ml (median: 0.961 ng/ml).

**Table 2:** 

Disease Course	Number	Age (range)	Gender	EDSS-Score	GFAP (ng/ml; range)
Secondary-chronic progressive MS	n = 4	25–55	3 F, 1 M	5.0–8.5	2.05 (1.7–2.4)
Relapsing-remitting MS	n = 10	19–55	6 F, 4 M	2.0–3.5	0.815 (0.587–1.275)
Non neurological disease	n = 8	18–44	4 F, 4 M	n.a.	0.961 (0.507–1.595)

### Proteome profiling in a disease model with enhanced tissue damage

**Figure 4:**
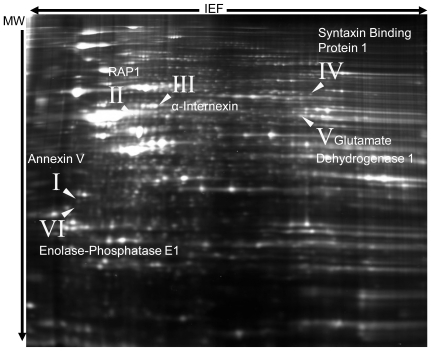
Proteome analysis of CNTF knockout mice. 2D-DIGE gel of spinal cord proteins from CNTF knock-out mice with EAE, labeled with Cy3 (shown in green) versus wild type mice with EAE labeled with Cy5 (shown in red). Selected proteins identified by Mass Spectrometry are indicated with roman indices (see Fig. 5). Proteins were separated on 24 cm IPG Strip pH 3–10 non-linear.

**Figure 5:**
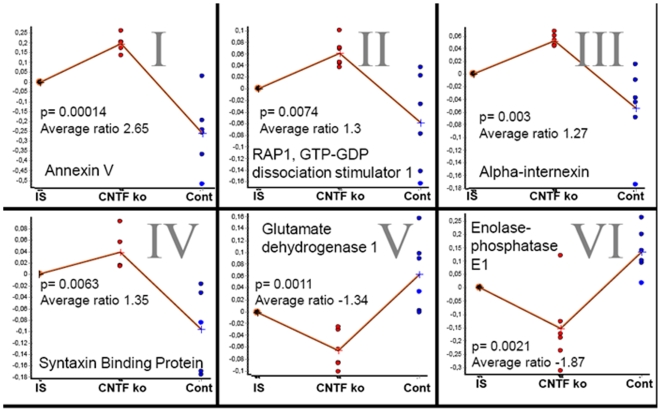
Statistical evaluation of regulated proteins in CNTF −/− mice. 2D DIGE analysis of the average spot volume increase ratio of proteins shown in Fig. 4. Using the DeCyder's Biological Variation Analysis module, a paired Student's t-test yielded a p-value within the 99th percentile confidence level. Mean value crosses are connected; IS  =  Internal Standard.

Finally, we were interested to investigate marker proteins not only correlating with the disease stage, but also patterns of tissue damage. Thus, we investigated the regulation of proteins after induction of MOG-EAE in the model of the CNTF −/− mouse, a well characterized paradigm with enhanced OL apoptosis and axon damage on days 28 and 56 p.i. after immunization [25]. Proteome profiling of the early chronic phase of MOG-EAE in CNTF −/− versus wild-type mice was performed on day 37 p.i. At this time, CNTF −/− mice displayed a more pronounced motor impairment as compared to matched controls (mean clinical score ± SEM: 6.1±0.6 in CNTF −/− mice vs. 4.6±0.7 in wild-type mice, p = 0.02). Proteomic analyses revealed a number of significantly regulated proteins (Table 3) as proven by analysing groups of individual mice. While this approach did not reveal the regulation of the stage-specific marker GFAP as associated with enhanced tissue damage, the analysis disclosed an increase in the axonal structure proteins alpha-internexin as part of the neurofilament apparatus and also syntaxin binding protein 1 in CNTF −/− mice (see Fig. 4 for gel and Fig. 5 for quantitative analysis). Moreover, CNTF −/− mice displayed an increase in annexin V as apoptosis marker, but a decrease in glutamate decarboxylase which is involved in glutamate metabolism and and also altered in demyelinated lesions [35]. Finally, we identified an increase in RAP1 (GTP-GDP dissociation stimulator 1) and a decrease of enolase-phosphatase 1 in CNTF −/− mice (see table 3) as proteins of the cellular metabolism with a so far miscellaneous function in EAE. These changes were not found in first analyses comparing naïve CNTF −/− and wild-type mice.

**Table 3:** 

Spot No.	Protein name	Swiss-Prot accession	MW [kDa]	pI	PMF coverage [%]	PMF score^a^	Peptide sequenced	MS/MS ion score^b^
I	Annexin A5	P48036	35.8	4.8	23	67	GTVTDFPGFDGR	27
							GLGTDEDSILNLLTSR	25
							FITIFGTR	6
II	RAP1, GTP-GDP dissociation stimulator 1	NCBI: gi|148680137^c^	61.1	5.4	59	177	DLASAQLVQILHR	73
							HVEDGNVTVQHAALSALR	58
III	Alpha-internexin	P46660	55.9	5.2	50	242	HSAEVAGYQDSIGQLESDLR	139
							FSTGGLSISGLNPLPNPSYLLPPR	99
IV	Syntaxin-binding protein 1	O08599	67.9	6.5	26	82	DNALLAQLIQDK	14
							LAEQIATLCATLK	14
VI	Enolase-phosphatase E1	Q8BGB7	28.7	4.8	21	32	AEFFADVVPAVR	44
							LLFGHSTEGDILELIDGHFDTK	6
V	Glutamate dehydrogenase 1, mitochondrial precursor	P26443	61.6	8.1	48	162	HGGTIPVVPTAEFQDR	54
							IIKPCNHVLSLSFPIR	50

a) Mascot protein score obtained for the peptide mass fingerprint (PMF). The significance threshold was 54.

b) Mascot MS/MS ion scores obtained for the individual peptides sequenced. The significance threshold for identity was 22–28 depending on how many peptides fell within the mass tolerance window about the precursor mass. Only the top ranking peptides matching a query for the first time (“bold red hits”) are reported.

c) Data were searched against the NCBI data base as the search against the Swiss-Prot database did not reveal any identification.

**Figure 6:**
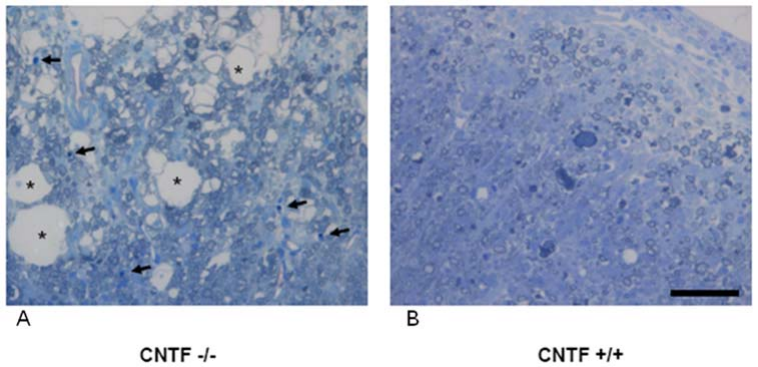
CNTF deficiency leads to destructive EAE lesions with enhanced apoptosis. EAE lesions in CTNF −/− mice (left) are destructive with vacuolar degeneration (asterisks) and multiple apoptotic cells with pycnotic nuclei (depicted by arrows). These changes are not present in EAE lesions from wild-type control mice (right side). Representative cervical spinal cord cross sections from a CNTF −/− knockout mouse and a wild-type CNTF +/+ control mouse on day 35 p.i. of MOG-EAE are shown. Bar  = 50 µm.

In summary, the regulated proteins point at an increased apoptosis and glutamate mediated excitotoxicity along with changes in the axonal neurofilament apparatus in EAE diseased CNTF −/− mice. These observations correlate well with the destructive pathology and also OL apoptosis in EAE lesions of CNTF deficient mice on day 35 p.i. (Fig. 6) which were already described on days 28 and 56 p.i. in earlier studies [25].

## Discussion

Here we performed proteome profiling of murine MOG-EAE as a model of secondary progressive MS.

First, our data reveal an up-regulation of GFAP in the spinal cord in the chronic disease phase of MOG-EAE. These findings could be transferred from the experimental model to the clinical setting with an increase of GFAP also in the CSF of secondary progressive MS patients. An increase of GFAP expression during autoimmune demyelination was already shown in different models of EAE [36]–[38] with acute as well as relapsing remitting disease courses. Our data extend these previous findings also to the chronic phase of MOG-EAE. The up-regulation of GFAP in the later phase of the disease implies a role of astroglia during chronicity of autoimmune demyelination. In contrast, some previous studies also point towards a role of glial cells in the regulation of the target tissue response to an inflammatory insult [39]. Here, studies in the model of GFAP knockout mice are somewhat hampered by a complex phenotype with impaired white matter architecture [40].

Moreover, previous data indicate that assessment of GFAP in the CSF of MS patients with a newly developed ELISA may be feasible [41]. Indeed, an increase of GFAP was also found in the CSF of MS patients, in particular in secondary progressive MS patients which is well in line with previous observations [37], [38] and adds GFAP to other markers like neurofilament or nitric oxide metabolites that were also shown to correlate with disability or disease progression [42], [43].

The similar findings on the regulation of GFAP in spinal cord tissue of MOG-EAE and the CSF from secondary progressive MS patients speak for a good correlation of changes in protein expression in the CNS from the animal model and the CSF compartment in MS patients. Moreover, these data also serve as a proof of principle revealing that proteomics can be a valuable tool for translational medicine. Proteome analysis of murine EAE models may allow the identification of candidate biomarkers which can then be transferred to the respective MS stages in clinical practice. Indeed, CNS derived proteins may not only serve as bulk markers of the disease [44], but could also show stage specificity thus helping to predict disease prognosis.

Second, proteome analysis of MOG-EAE in CNTF −/− mice reveals a regulation of proteins involved in apoptosis (annexin V) and also regulation of excitoxicity (glutamate decarboxylase). Moreover, CNTF −/− mice displayed an increase in axonal structure proteins like alpha-internexin indicating an enhanced axonal disintegration. These findings correlate well with the previously described histopathological pattern in CNTF −/− mice which includes an increased OL apoptosis and also an enhanced axonal injury. In the light of the decreased expression of glutamate decarboxylase in CNTF −/− mice and the role of glutamate excitotoxicity as a mechanism for axonal damage and OL death in autoimmune demyelination [45], it is tempting to speculate that a decreased glutamate metabolism may lead to enhanced excitotoxicity which then precipitates the pronounced tissue damage in this model.

Other candidate markers which are possibly linked to pathomechanisms of disease and were identified in specimen of MS patients include hypoxia associated factors [46] for ischemic tissue damage, or, most recently identified, neurofascin for axonal injury [47]. As identified in CNTF −/− mice, alpha internexin may present another interesting candidate marker for degenerative changes in MS, similar to neurofilament proteins (see above).

In summary, our findings underscore the value of proteome profiling as screening technique for biomarkers determining the disease stage, but also for the identification of prevailing pathomechanisms in autoimmune demyelination. In particular, stage specific EAE models [19] may indicate which molecule bears the highest level of success as biomarker for disease progression not only for EAE itself, but possibly also for MS. While proteomics may help to identify new biomarkers, this technique could also be beneficial to understand pathophysiological changes or to predict treatment responses in chronic inflammatory diseases of the CNS as recently shown for DNA vaccination [18].
